# Prognostic Impact of Immune-Inflammatory and Nutritional Indices in Metastatic Hormone-Sensitive Prostate Cancer

**DOI:** 10.3390/diagnostics16132126

**Published:** 2026-07-07

**Authors:** Mehmet Nuri Baser, Ahmet Baklaci, Ahmet Unlu, Asim Armagan Aydin, Zeynel Umut Alpsoy, Suleyman Utku Uzun, Bilgin Demir

**Affiliations:** 1 Department of Medical Oncology, Faculty of Medicine, Aydin Adnan Menderes University, 09100 Aydin, Turkey; mbaser@adu.edu.tr (M.N.B.); baklaci.ahmet@gmail.com (A.B.); 2 Department of Medical Oncology, University of Health Sciences, Antalya Education and Research Hospital, 07100 Antalya, Turkey; md.ahmetunlu@gmail.com (A.U.); drarmaganaydin@gmail.com (A.A.A.); 3 Department of Internal Medicine, Faculty of Medicine, Aydin Adnan Menderes University, 09100 Aydin, Turkey; umut.alpsoy@hotmail.com; 4 Epidemiology Division, Department of Public Health, Faculty of Medicine, Pamukkale University, 20070 Denizli, Turkey; suzun@pau.edu.tr

**Keywords:** metastatic hormone-sensitive prostate cancer, CALLY index, systemic inflammation, prognosis, immune-inflammatory markers

## Abstract

**Background/Objectives**: Systemic inflammation and nutritional status influence cancer progression and prognosis. The C-reactive protein–albumin–lymphocyte (CALLY) index is a novel biomarker reflecting inflammation, immune response, and nutritional status; however, its prognostic value in metastatic hormone-sensitive prostate cancer (mHSPC) remains unclear. **Methods**: This retrospective multicenter cohort study included 159 patients with de novo mHSPC diagnosed between January 2018 and April 2025. Baseline CALLY, neutrophil-to-lymphocyte ratio (NLR), systemic immune-inflammation index (SII), and systemic inflammation response index (SIRI) were calculated. **Results**: The optimal CALLY cut-off was 0.58. A low CALLY was associated with significantly shorter progression-free survival (PFS) and overall survival (OS). It correlated with higher tumor burden, higher Gleason grade, elevated prostate-specific antigen levels, and poorer performance status. In univariate analysis, a low CALLY predicted worse OS (HR 5.20; *p* = 0.021), although this effect was attenuated in multivariate analysis (HR 2.15; 95% CI 0.52–8.90; *p* = 0.290), and its absolute discriminatory performance in receiver operating characteristic analysis remained modest (AUC = 0.558). Complete response rates differed significantly (high-CALLY 41.7% vs. low-CALLY 5.9%, *p* < 0.001), suggesting a potential link between baseline CALLY and treatment response. **Conclusions**: Among the indices evaluated, CALLY demonstrated the highest, though still modest, discriminatory ability for overall survival (AUC 0.558), and was the only marker to reach statistical significance in survival analysis. Its prognostic effect was attenuated in multivariable analysis, suggesting that CALLY reflects, rather than independently drives, the systemic consequences of high-burden disease. These findings are exploratory, and prospective validation in larger cohorts is required to determine its potential clinical utility.

## 1. Introduction

Prostate cancer (PC) is the most commonly diagnosed malignancy among men, accounting for approximately 31% of all newly diagnosed cancers, with an estimated 333,830 new cases and 36,320 deaths expected in the United States in 2026 [1]. Metastatic prostate cancer (mPC), being a heterogeneous group of diseases, exhibits varying responses to treatment and can progress from the oligometastatic stage to advanced disease [2]. Treatment for metastatic hormone-sensitive prostate cancer (mHSPC) commences with androgen deprivation therapy (ADT) and androgen receptor pathway inhibitors (ARPIs) [3]. A substantial percentage of patients exhibit a response to these treatments; however, the majority advance to a castration-resistant phase [4]. This situation creates a need for reliable prognostic biomarkers to reassess risk stratification and better manage the treatment decision-making process.

Increasing evidence suggests that systemic inflammatory processes that damage deoxyribonucleic acid (DNA) and activate oncogenic pathways play a role in the development and progression of many cancers, including PC [5,6].

Several immuno-inflammatory markers derived from standard blood tests, including the neutrophil-to-lymphocyte ratio (NLR); the systemic immune-inflammation index (SII) incorporating neutrophil, platelet, and lymphocyte counts; and the systemic inflammation response index (SIRI) comprising monocyte, neutrophil, and lymphocyte values, have exhibited prognostic significance in investigations of mPC [7]. The C-reactive protein (CRP)–albumin–lymphocyte (CALLY) index has recently been identified as a new prognostic biomarker for many cancer types, offering a thorough evaluation of the patient’s immunological response and nutritional condition [8,9,10]. The functional significance of the CALLY index within mPC remains undetermined. We hypothesized that the CALLY index, which assesses systemic inflammation, nutritional status, and immune function together, might provide useful prognostic information compared with other established important immune-inflammatory markers in de novo mHSPC patients and that a low CALLY index might be associated with poorer survival outcomes.

Therefore, the aim of this study is to investigate the prognostic value of the CALLY index in conjunction with other immune-inflammatory indices and to evaluate its potential association with clinicopathological features and survival outcomes in de novo mHSPC patients. This may potentially contribute to improving risk stratification, identifying high-risk patients, and developing more individualized treatment strategies.

## 2. Materials and Methods

### 2.1. Study Design and Ethics

This study was structured as a multicenter retrospective observational cohort analysis involving patients with de novo mHSPC treated at the Medical Oncology Departments of Aydin Adnan Menderes University Hospital and Antalya Training and Research Hospital. The protocol for the study was approved by the Institutional Review Board of Aydin Adnan Menderes University, and it was conducted in accordance with the ethical standards outlined in the Declaration of Helsinki (Date: 7 November 2025; Decision No: 28-2025/341).

### 2.2. Patient Selection

In the period beginning in January 2018 and ending in April 2025, we carried out a multicenter retrospective analysis of the medical records of patients who had been diagnosed with de novo mHSPC. The participants in the research were 159 patients who had been diagnosed with histologically proven PC and radiographically confirmed metastatic disease at the time of examination. Participants were required to be at least 18 years old and have a minimum follow-up time of six months in order to be eligible. Every patient who took part in the study was subjected to first-line intensified ADT, which consisted of medical castration (leuprorelin acetate 22.5 mg every three months or 45 mg every six months; goserelin 3.6 mg monthly or 10.8 mg every three months) in conjunction with ARPIs such as abiraterone acetate (1000 mg/day), apalutamide (240 mg/day), or enzalutamide (160 mg/day).

**Figure 1:**
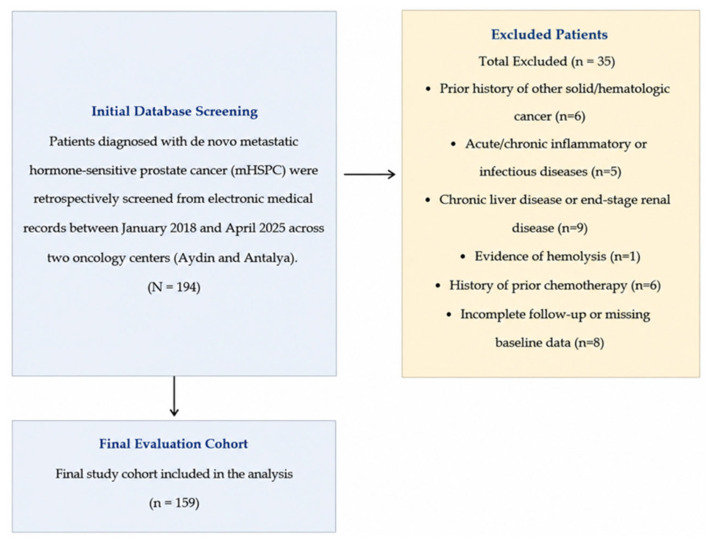
Flow diagram of patient selection and eligibility.

Patients who had a history of chemotherapy or any other solid or hematologic malignancy, as well as acute or chronic inflammatory or infectious diseases, chronic liver disease, end-stage renal disease, or evidence of hemolysis, were excluded from the study. Patients with incomplete follow-up data were also omitted from the final cohort (Figure 1). Baseline laboratory data were obtained within 14 days prior to treatment initiation.

### 2.3. Data Collection and Definitions

Baseline clinicopathological parameters, such as age, Eastern Cooperative Oncology Group (ECOG) performance status, Gleason score, and patterns of metastasis, were extracted from electronic medical records. Tumor burden and risk stratification were defined according to established pivotal trial criteria:

- CHAARTED Volume Criteria: High-volume disease was defined as the presence of visceral metastases or ≥4 bone lesions with at least one outside the vertebral column and/or pelvis [11].
- LATITUDE Risk Criteria: High-risk disease was defined as meeting at least two of the following three criteria: (1) Gleason score ≥ 8, (2) presence of ≥3 bone lesions, and (3) presence of measurable visceral metastasis [12].

### 2.4. Calculation of CALLY Index and Inflammatory Indices

The primary prognostic variable, the CALLY Index, was calculated using the following formula:

CALLY Index = [Serum albumin level (g/dL) × absolute lymphocyte count (ALC) (10^9^/L)]/[CRP (mg/L) × 10]

For comparative analysis, other immune-inflammatory indices were calculated as follows:

- Neutrophil-to-Lymphocyte Ratio (NLR): absolute neutrophil count (ANC)/ALC
- Systemic Immune-Inflammation Index (SII): [(Platelet × ANC)]/ALC
- Systemic Inflammation Response Index (SIRI): [ANC × absolute monocyte count (AMC)]/ALC

### 2.5. Outcome Measures

This study’s main outcome was overall survival (OS). OS was assessed as the amount of time that passed between the initial diagnosis and the death that occurred owing to any cause. An additional end point was defined as progression-free survival (PFS) in this study. PFS was defined as the time from diagnosis to first radiographic progression or death from any cause, whichever occurred first. Progression of PSA or isolated clinical progression without radiographic confirmation was not included in the definition of PFS. Given the molecular nature of the imaging, radiographic progression was objectively assessed by ^68^Ga-PSMA PET/CT imaging using the Prostate Cancer Working Group 3 (PCWG3) guidelines and PSMA PET Progression (PPP) criteria (e.g., the appearance of new, unambiguous PSMA-avid lesions). Best overall tumor response was assessed according to the Response Evaluation Criteria in PSMA PET/CT (RECIP 1.0). The first response assessment was performed approximately 12 weeks after treatment initiation, and the best overall response was defined based on serial ^68^Ga-PSMA PET/CT scans obtained before radiographic progression. During the follow-up period, patients were routinely evaluated with clinical and imaging evaluation approximately every 3–6 months or earlier in case of clinical progression or significant PSA rise. Patients were censored in the case of being alive and had no disease progression at their last follow-up date.

### 2.6. Statistical Analysis

All statistical analyses were performed with the R statistical program (version 4.5.2; R Foundation for Statistical Computing, Vienna, Austria). Continuous data were tested for normality. Continuous data were described using median and interquartile range (IQR) or mean and standard deviation (SD). Variables were evaluated using the Kruskal–Wallis test or analysis of variance (ANOVA) according to the normality distribution. Categorical data were expressed as percentage and frequency. Categorical variables were analyzed using Pearson’s chi-squared test or Fisher’s exact test. The cut-off value that gives the highest level of precision in terms of progression and survival was found with the application of Receiver Operating Characteristic (ROC) curve analysis utilizing Youden’s J statistic. Survival curves were constructed using the survminer program (version 0.4.9). The estimates of OS and PFS were obtained using the Kaplan–Meier method. Survival rates between the groups were compared using the log-rank test. Multivariate and univariate Cox proportional hazards models were constructed using the survival package (version 3.5-7) to evaluate independent prognostic markers. We examined scaled Schoenfeld residuals to assess the proportional hazards assumption. Statistical significance was recognized when the *p* value was <0.05 on both sides.

To respect the events-per-variable (EPV) criterion of ≥10, which yielded a maximum of four variables in the multivariable model (41 events ÷ 10 = 4.1), variables for inclusion were pre-selected based on clinical significance and a priori confounding potential. CHAARTED volume and Gleason grade were selected for their established prognostic relevance; age (>65 years) was included as an a priori confounder. The proportional hazards assumption was tested for all Cox models using scaled Schoenfeld residuals (cox.zph function). In terms of the variable selection rationale, LATITUDE risk was excluded due to collinearity with CHAARTED volume (shared defining parameters: bone lesion count, Gleason grade, visceral metastasis). Visceral metastasis was excluded, as it is a component of the CHAARTED high-volume definition. ECOG performance status was excluded because it is conceptually and statistically correlated with the CALLY index (both capture functional and nutritional reserve). Baseline PSA was excluded, as it is partly captured by tumor volume classification.

To confirm the robustness of the categorical findings, the CALLY index was evaluated as a continuous predictor variable. The linear continuous relationship was modeled to estimate hazard ratios per 0.1-unit and per 1-standard deviation (SD) increases for OS and PFS. To assess potential non-linearity in the CALLY–OS relationship, a natural cubic spline model with 3 degrees of freedom was constructed and evaluated using a likelihood-ratio test. Internal validation and stability of the data-driven Youden-derived threshold were assessed using bootstrap resampling with 1000 iterations to estimate the mean concordance index (C-index) and bootstrap median cutoffs with corresponding 95% confidence intervals.

## 3. Results

A total of 159 individuals with mHSPC were included in this retrospective study. The median age of the patients was 72.7 years ([IQR], 64.9–77.7 years). Half of the patients had CHAARTED high-volume illness, and almost 60% were at high risk for LATITUDE. Enzalutamide (*n* = 78), abiraterone (*n* = 71), or apalutamide (*n* = 10) in conjunction with ADT constituted the treatment. The prognostic value of the CALLY index was evaluated using ROC curve analysis, with OS as the main outcome. With an ideal cut-off value of 0.58 determined using Youden’s J statistic, the ROC analysis yielded an AUC of 0.558 (95% CI, 0.46–0.66), which corresponds to a sensitivity of 95.1% and a specificity of 18.6%. Patients were divided into two groups based on this cut-off: one with low CALLY levels (CALLY ≤ 0.58; *n* = 135, 84.9%) and another with high CALLY levels (CALLY > 0.58; *n* = 24, 15.1%). The predominance of Low-CALLY patients (84.9%) is consistent with the heavy inflammatory burden typical of de novo metastatic presentation. The small High-CALLY subgroup (*n* = 24) reduces the precision of subgroup-specific estimates; confidence intervals for this group should be interpreted accordingly.

### 3.1. Baseline Characteristics

The research cohort’s baseline clinicopathological features are displayed in Table 1. A higher CALLY index was substantially correlated with more advantageous demographic and clinical attributes. Individuals in the Low-CALLY cohort were markedly older than those in the High-CALLY cohort (median age: 73.2 vs. 65.7 years; *p* = 0.009). The performance status varied markedly between groups, with a greater percentage of patients exhibiting ECOG PS ≥ 2 in the low-CALLY group (22.2% vs. 12.5%, *p* = 0.414).

Concerning disease features, patients in the low-CALLY group had markedly elevated frequencies of Gleason Grade Group 4–5 (86.7% vs. 58.3%, *p* = 0.003), CHAARTED high-volume disease (63.7% vs. 33.3%, *p* = 0.010), and LATITUDE high-risk categorization (66.7% vs. 33.3%, *p* = 0.004). Additionally, baseline prostate-specific antigen (PSA) levels were significantly higher in the low-CALLY group (median 86.1 vs. 16.4 ng/mL, *p* = 0.003). In the high-CALLY group, the complete response rate was much greater (43.5% vs. 6.1%, *p* < 0.001).

The prevalence of hypertension (HT) (64.4% vs. 54.2%, *p* = 0.465), diabetes mellitus (DM) (37.8% vs. 41.7%, *p* = 0.894), coronary artery disease (CAD) (34.1% vs. 33.3%, *p* = 1.000), statin use (40.0% vs. 45.8%, *p* = 0.756), and aspirin use (37.8% vs. 41.7%, *p* = 0.894) showed no statistically significant differences across the categories. Notably, baseline comorbidities and concomitant medications—including statin and aspirin use—did not differ significantly between CALLY groups (all *p* > 0.05, Table 1), arguing against major confounding by these variables in the primary survival analysis. Also, tumor burden was significantly inversely associated with the CALLY index. Among patients categorized as High-CALLY, there was a statistically significant increase in the prevalence of low-volume illness (66.7% vs. 36.3%, *p* = 0.010) and low-risk disease (66.7% vs. 33.3%, *p* = 0.004). In terms of visceral metastatic status (*p* = 0.181), there were no statistically significant differences between the groups. Notably, the type of first-line ARPI did not differ significantly between CALLY groups (*p* = 0.300, Table 2), indicating that ARPI-class selection does not confound the primary survival analysis.

### 3.2. Survival Outcomes

**Figure 2:**
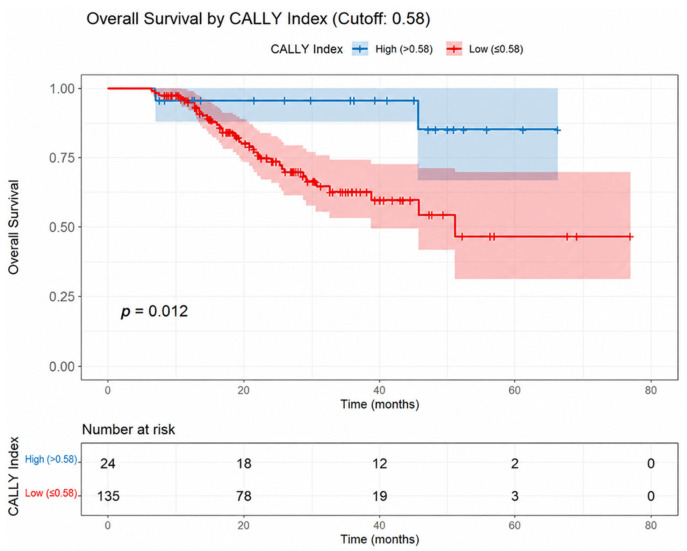
**Overall Survival (OS) According to CALLY Index Status.** CALLY index, C-reactive protein–albumin–lymphocyte index.

**Figure 3:**
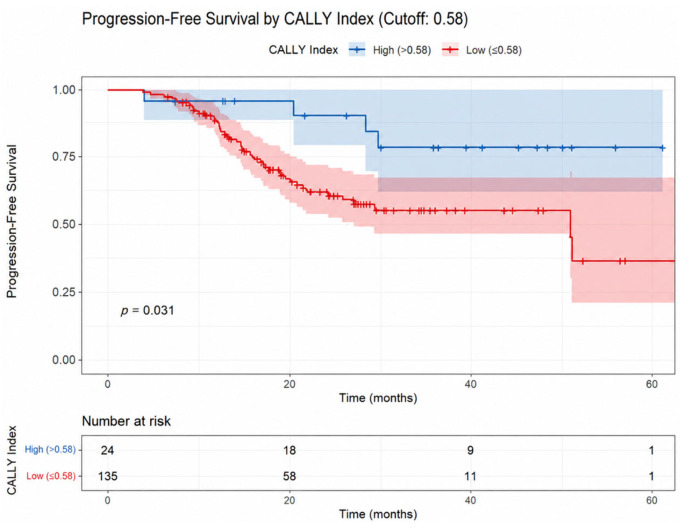
**Progression-Free Survival (PFS) According to CALLY Index Status.** CALLY index, C-reactive protein–albumin–lymphocyte index.

After the follow-up period ended, disease progression was observed in 47 patients (34.8%) in the Low-CALLY group and 4 patients (16.7%) in the High-CALLY group (chi-square *p* = 0.129). Among the 59 composite PFS events, 51 were confirmed radiographic progressions and 8 were deaths occurring without prior documented radiographic progression. Although isolated radiographic disease progression was observed in 51 patients, the total number of PFS events used in the Kaplan–Meier analysis was 59, as this composite endpoint also encompasses deaths prior to documented progression. The low-CALLY group also had 39 fatalities, or 28.9% of the total, whereas the high-CALLY group had 2 fatalities, or 8.3% of the total. The Kaplan–Meier survival estimates are shown in Figure 2 and Figure 3. The total group had a median PFS of 51.1 months (95% CI, 50.8-not attained). Patients in the Low-CALLY group had a median PFS of 50.8 months (95% CI, 27.0-not reached), but those in the High-CALLY group did not reach the median PFS (log-rank *p* = 0.031).

The whole study population did not achieve the median in terms of OS. Patients in the High-CALLY group outlived those in the Low-CALLY group by a significant margin; the median OS for the Low-CALLY group was 51.2 months (95% CI, 38.8-not reached), whereas the High-CALLY group did not reach the median OS (log-rank *p* = 0.012). Consistently, the cumulative mortality rate was higher in the Low-CALLY group compared to the High-CALLY group (28.9% [39 fatalities] vs. 8.3% [2 fatalities], chi-square *p* = 0.062).

### 3.3. Prognostic Factors and Comparative Analysis

Univariate and multivariate Cox proportional hazards regression analysis were performed to uncover independent prognostic indicators for OS (Table 3). There was a significant association between a low CALLY index (≤0.58) and an elevated risk of all-cause death (hazard ratio [HR], 5.20; 95% confidence interval [CI], 1.25–21.6; *p* = 0.021) on univariate analysis. Furthermore, significant predictors of shortened survival were high-volume disease as determined by the CHAARTED criteria (HR 4.87, *p* = 0.001) and raised Gleason scores (HR 1.89, *p* = 0.014). High tumor volume was the most significant independent predictor of OS in the multivariate model that controlled for age, Gleason score, CHAARTED-defined tumor volume, and CALLY index (HR 4.47, 95% CI 1.74–11.45; *p* = 0.002). Table 3 shows that multivariate analysis reduced the predictive power of the CALLY index (HR 2.15; *p* = 0.290).

**Figure 4:**
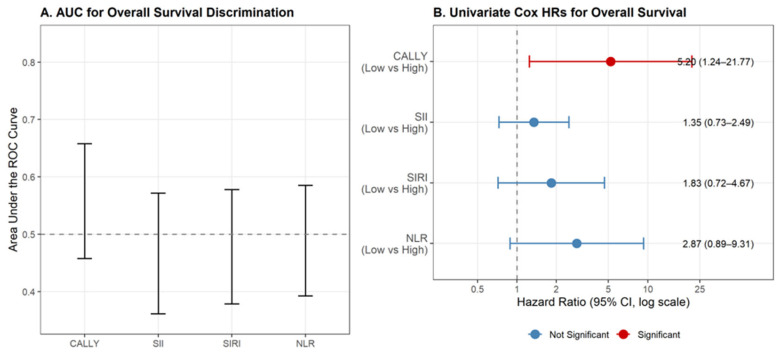
**Comparative Performance of Immune-Inflammatory and Nutritional Indices for OS Prediction.** (**A**) Area under the ROC curve (AUC) with 95% confidence intervals for OS event-status discrimination. All AUCs were modest; CALLY demonstrated the numerically highest value. (**B**) Forest plot of univariate Cox regression hazard ratios (95% CIs) for indices dichotomized at their respective Youden-derived thresholds, with the high-value group as reference for all markers. Non-significant hazard ratios for SII, SIRI, and NLR reflect heavily imbalanced group sizes at these thresholds and should not be interpreted as indicating superior outcomes for high inflammatory burden. The original four-curve ROC plot is provided in Supplementary Figure S1.

A comparative ROC analysis was carried out to evaluate the CALLY index’s discriminative performance in comparison to other recognized indicators of systemic inflammation (Table 4). Among the four indices, CALLY demonstrated the numerically highest discriminatory ability (AUC 0.558), though all AUCs were modest, with 95% CIs overlapping 0.50 (Table 4, Figure 4). In terms of OS prediction accuracy, the CALLY index was the most effective (AUC 0.558), surpassing SII (AUC 0.534), SIRI (AUC 0.522), and NLR (AUC 0.511). Importantly, CALLY was the only marker to reach statistical significance for OS (*p* = 0.012) (Table 4).

### 3.4. Sensitivity Analyses

When analyzed as a continuous variable, each 0.1-unit increase in the CALLY index was associated with a non-significant trend toward reduced hazard of death (univariate HR = 0.915, 95% CI 0.822–1.019, *p* = 0.107) and progression (univariate HR = 0.933, 95% CI 0.862–1.009, *p* = 0.084), confirming the protective direction of the categorical analysis. Per 1-SD increase (1 SD = 1.46), the univariate OS HR was 0.275 (95% CI 0.057–1.319, *p* = 0.107). A natural cubic spline model (3 degrees of freedom) did not demonstrate significant non-linearity in the CALLY–OS relationship (likelihood-ratio test: χ^2^ = 0.166, df = 2, *p* = 0.920; Supplementary Figure S2), supporting the use of CALLY as a linear continuous predictor. The spline curve should be interpreted cautiously at CALLY values above 1.0, as only 2 deaths occurred in the high-CALLY group (*n* = 24) and confidence intervals widen substantially due to sparse data. Bootstrap resampling (1000 iterations) yielded a mean C-index of 0.595 (95% CI 0.497–0.686) for the CALLY-only Cox model, consistent with the apparent C-index of 0.696 for the full multivariable model. The Youden-derived cutoff of 0.58 showed a bootstrap median of 0.046 (95% CI: 0.004–0.907), indicating considerable instability of the cutpoint across resamples (Supplementary Figure S4); this underscores the importance of reporting continuous hazard ratios alongside data-driven cutpoints.

## 4. Discussion

With an expected increase in incidence over the next decade, PC remains the second most frequently diagnosed malignancy among men worldwide [13]. Increasing evidence suggests that systemic inflammation and nutritional status play a central role in cancer progression and survival outcomes. The CALLY index, a recently developed composite biomarker integrating serum albumin, lymphocyte count, and C-reactive protein, reflects the interaction between nutritional reserve, systemic inflammation, and host immune competence. To our knowledge, this is the first multicenter study evaluating the prognostic significance of the CALLY index in patients with mHSPC. In our cohort, the CALLY index demonstrated a numerically stronger association with survival outcomes than conventional inflammatory indices such as NLR, SII, and SIRI. However, its prognostic effect attenuated after adjustment for established disease-related variables, suggesting that CALLY may primarily reflect tumor burden and host-related biological status rather than functioning as an entirely independent prognostic determinant.

The attenuation of the CALLY association with overall survival after adjustment for CHAARTED-defined tumor volume and Gleason score (HR 2.15; 95% CI 0.52–8.90; *p* = 0.290) indicates that a substantial part of the prognostic information captured by CALLY at the univariate level (HR 5.20; *p* = 0.021) overlaps with established measures of disease extent. CHAARTED volume emerged as the dominant independent predictor (HR 4.47; *p* = 0.002), consistent with its established role in contemporary mHSPC management. This pattern suggests that CALLY reflects the systemic inflammatory and nutritional consequences of advanced tumor biology rather than an independent causal pathway, consistent with the degree of HR attenuation observed in multivariable analysis. This does not, however, preclude its potential exploratory utility: CALLY provides a rapid, routinely available blood-based summary of host physiology that is not substitutable by imaging criteria and may complement staging in resource-limited settings.

Importantly, although the CALLY index showed numerically higher discriminatory performance than NLR, SII, and SIRI in ROC analyses, the absolute differences between the indices were small, and all evaluated inflammatory markers showed only modest discriminatory ability (AUC range: 0.511–0.558). This baseline limitation is frequently encountered in de novo mHSPC cohorts, where high systemic tumor burden and profound inflammatory heterogeneity complicate sharp cross-sectional separation. Therefore, these findings should be interpreted as demonstrating a relative advantage rather than a definitive standalone diagnostic solution of the CALLY index and should be considered strictly exploratory and hypothesis-forming until external validation is performed in larger prospective studies.

Chronic inflammation is recognized as a major contributor to prostate carcinogenesis and progression. Persistent inflammatory stimulation induced by hormonal, environmental, infectious, or chemical factors promotes recruitment of immune cells into the tumor microenvironment and facilitates tumor development [14]. Elevated inflammatory markers, including CRP and hematologic parameters such as white blood cell count, have consistently been associated with adverse oncologic outcomes, while lymphocytes play a critical role in antitumor immune surveillance [15,16]. Previous studies demonstrated the prognostic relevance of inflammatory indices in mHSPC. Wang et al. identified NLR, SII, and SIRI as independent predictors of castration-free survival and OS, with elevated levels correlating with poorer outcomes [7]. Similarly, in localized prostate cancer treated with radical prostatectomy, higher NLR and SII values were associated with more aggressive pathological characteristics [17].

In the present study, NLR, SII, and SIRI did not retain independent prognostic significance in multivariable analyses. This finding may partly reflect the limited sample size and event rate of our cohort, which inherently limits the capability to detect modest independent survival effects for these conventional cellular markers within a highly adjusted model. In addition, variability in optimal cut-off values across cohorts may have contributed to these findings. The relatively higher SII threshold observed in our study compared with previous reports likely reflects the increased baseline inflammatory burden associated with de novo metastatic disease presentation. Given the absence of universally accepted cut-off values for inflammatory indices, future multicenter studies should prioritize standardized thresholds or continuous effect modeling to improve reproducibility across cohorts.

Cancer-associated malnutrition and systemic inflammation are well-established determinants of survival outcomes. Although mPC demonstrates lower malnutrition rates than many other advanced malignancies [18], hypoalbuminemia remains a recognized marker of poor prognosis [19]. By combining albumin, lymphocyte count, and CRP into a single parameter, the CALLY index simultaneously captures nutritional, inflammatory, and immunological status. Mechanistically, elevated CRP reflects an IL-6–driven acute-phase response associated with STAT3-mediated tumor progression and immune suppression, whereas hypoalbuminemia represents chronic inflammatory catabolism and reduced physiologic reserve [20]. Lymphopenia, in contrast, indicates impaired adaptive immunity and diminished cytotoxic T-cell activity [16]. In prostate cancer specifically, serum albumin levels are often relatively preserved compared with highly cachectic malignancies, suggesting that inflammatory activation and lymphocyte depletion may drive reductions in CALLY scores before overt malnutrition becomes clinically apparent. By combining these kinetics, the CALLY index captures the intricate interplay between an IL-6–driven acute-phase response and systemic nutritional decline. This multi-dimensional biological structure explains why it retains a numerical advantage in prognostic utility over purely cellular parameters (NLR, SII, SIRI) when assessing long-term OS, as tumor-induced metabolic exhaustion heavily dictates the clinical course of advanced prostate cancer. Our findings further supported the biological relevance of systemic inflammation in advanced prostate cancer. Di Lorenzo et al. previously demonstrated that patients with progressive prostate cancer frequently exhibited severe lymphopenia and elevated CRP levels [21]. Similarly, the predominance of low CALLY scores in our mHSPC cohort suggested that a subset of patients may already display a castration-resistant prostate cancer (CRPC)-like inflammatory and immunosuppressive phenotype at initial diagnosis. This observation may partly explain the poorer outcomes observed in these patients and suggested that the CALLY index could serve as an exploratory biological indicator of aggressive disease biology.

The prognostic significance of the CALLY index has previously been demonstrated across multiple malignancies, including hepatocellular, gastric, esophageal, breast, lung, colorectal, oral cavity, nasopharyngeal, and hypopharyngeal cancers [8,9,10,22,23,24,25,26,27]. Consistent with these reports, low CALLY scores in our cohort were significantly associated with inferior PFS and OS. Additionally, low CALLY scores correlated with advanced age, poorer ECOG performance status, higher PSA levels, increased Gleason grade, and high-volume disease according to CHAARTED criteria. These associations support the concept that the CALLY index reflects both tumor aggressiveness and deterioration of host physiological reserve. Importantly, CHAARTED volume (HR: 4.47, *p* = 0.002) and Gleason score (HR: 1.83, *p* = 0.024) remained independent predictors of survival in multivariable analyses, reinforcing the methodological consistency of our findings with established prognostic models such as CHAARTED and LATITUDE.

Notably, the association between low CALLY scores and reduced complete response rates to ARPI therapy raised the possibility that the index may also reflect treatment sensitivity. However, given the relatively limited discriminating ability demonstrated in ROC analyses, the CALLY index presently cannot be interpreted as a single predictive biomarker of treatment response and requires prospective validation of its potential predictive utility. Patients with preserved nutritional and immune status may maintain a more favorable antitumor microenvironment that enhances responsiveness to androgen receptor pathway inhibition. Although exploratory, this observation is clinically relevant given the current lack of validated predictive biomarkers for ARPI efficacy in mHSPC. Previous meta-analytic data showed that conventional clinical parameters, including Gleason score, PSA level, and ECOG performance status, do not consistently predict ARPI benefit [28]. In this context, the CALLY index may provide complementary biological information by integrating systemic inflammation and host immune competence into a single readily accessible biomarker. However, these findings require prospective validation in larger cohorts with predefined treatment-response endpoints.

Several limitations of this study should be acknowledged. The retrospective design and limited sample size reduced the generalizability of the findings. The marked imbalance between low- and high-CALLY groups also limited subgroup analyses. Although baseline comorbidity burden appeared balanced, detailed sensitivity analyses incorporating individual comorbidities and PSA nadir were not performed. Median OS was not reached during follow-up, indicating immature survival data and necessitating longer observation for definitive long-term survival assessment. The absence of standardized threshold values for inflammatory indices remains an important limitation affecting reproducibility across studies. Specifically, with 41 observed events, the study had limited post hoc power (~42% for HR = 2.15, two-sided α = 0.05) and was not adequately powered to detect modest effects for NLR, SII, or SIRI in multivariable models. Negative findings for these indices should be interpreted cautiously. The 24:135 group imbalance—while reflecting the biological distribution of nutritional–inflammatory status in de novo mHSPC—limits statistical power in the smaller subgroup and widens confidence intervals. Bootstrap resampling (1000 iterations) yielded a median cutoff of 0.046 (95% CI 0.004–0.907), indicating that the Youden-derived threshold of 0.58 is unstable across resamples; this instability is expected given the skewed CALLY distribution and strongly supports the reporting of continuous hazard ratios alongside data-driven cutpoints in future studies.

## 5. Conclusions

The CALLY index may represent a promising and easily obtainable biomarker reflecting the interaction between systemic inflammation, nutritional reserve, and immune competence in mHSPC. Although its prognostic impact appears closely linked to established measures of tumor burden and disease aggressiveness, low CALLY scores were consistently associated with inferior survival outcomes and reduced treatment response. These findings suggest that the CALLY index may serve as an exploratory surrogate marker providing preliminary, hypothesis-generating insights into adverse host–tumor biology. These results are to be viewed as strictly exploratory. Prospective, multicenter studies are required to confirm its potential clinical value and to determine the potential role of the CALLY index in future risk stratification before any clinical application can be considered. Future research should clarify the timing of baseline laboratory sampling relative to ARPI initiation, standardize sample handling protocols, and model CALLY both using Youden-derived thresholds and as a continuous (per-SD) variable to limit sensitivity to arbitrary thresholds. Reporting continuous hazard ratios alongside data-driven cutpoints will facilitate future meta-analyses and pooled threshold estimations across cohorts.

## Figures and Tables

**Table 1:** **Baseline Clinicopathological and Demographic Characteristics of Metastatic Hormone-Sensitive Prostate Cancer (mHSPC) Patients as Defined by the CALLY Index.** (Cut-off value: 0.58 determined by Youden Index).

Characteristic	Total (*n* = 159)	CALLY High > 0.58 (*n* = 24)	CALLY Low ≤ 0.58 (*n* = 135)	*p*-Value
**Demographics**				
Age, years, median (IQR)	72.7 (64.9–77.7)	65.7 (59.9–73.9)	73.2 (66.4–77.7)	**0.009**
**ECOG Performance Status (PS)**				
0–1, *n* (%)	126 (79.2)	21 (87.5)	105 (77.8)	0.414
≥2, *n* (%)	33 (20.8)	3 (12.5)	30 (22.2)	
**BMI Category**				
BMI < 18.5 kg/m^2^, *n* (%)	2 (1.3)	0 (0.0)	2 (1.5)	0.629
BMI 18.5–24.9 kg/m^2^, *n* (%)	83 (52.2)	11 (45.8)	72 (53.3)	
BMI ≥ 25 kg/m^2^, *n* (%)	74 (46.5)	13 (54.2)	61 (45.2)	
**Comorbidities & Medications**				
HT, *n* (%)	100 (62.9)	13 (54.2)	87 (64.4)	0.465
DM, *n* (%)	61 (38.4)	10 (41.7)	51 (37.8)	0.894
CAD, *n* (%)	54 (34.0)	8 (33.3)	46 (34.1)	1.000
Statin use, *n* (%)	65 (40.9)	11 (45.8)	54 (40.0)	0.756
Aspirin use, *n* (%)	61 (38.4)	10 (41.7)	51 (37.8)	0.894
Cancer family history, *n* (%)	12 (7.5)	1 (4.2)	11 (8.1)	0.794
**Disease Characteristics**				
**Gleason (ISUP) Grade Group, *n* (%)**				
Grade Group 1 (ISUP; Gleason score 3 + 3)	9 (5.7)	5 (20.8)	4 (3.0)	**0.003**
Grade Group 2 (ISUP; Gleason score 3 + 4)	1 (0.6)	0 (0.0)	1 (0.7)	
Grade Group 3 (ISUP; Gleason score 4 + 3)	18 (11.3)	5 (20.8)	13 (9.6)	
Grade Group 4 (ISUP; Gleason score 4 + 4/3 + 5/5 + 3)	62 (39.0)	6 (25.0)	56 (41.5)	
Grade Group 5 (ISUP; Gleason score 4 + 5/5 + 4/5 + 5)	69 (43.4)	8 (33.3)	61 (45.2)	
**M Stage, *n* (%)**				
M1a	25 (15.7)	3 (12.5)	22 (16.3)	0.231
M1b	126 (79.3)	21 (87.5)	105 (77.8)	
M1c	8 (5.0)	0 (0.0)	8 (5.9)	
**CHAARTED Volume, *n* (%)**				
Low volume	65 (40.9)	16 (66.7)	49 (36.3)	**0.010**
High volume	94 (59.1)	8 (33.3)	86 (63.7)	
**LATITUDE Risk, *n* (%)**				
Low risk	61 (38.4)	16 (66.7)	45 (33.3)	**0.004**
High risk	98 (61.6)	8 (33.3)	90 (66.7)	
**Visceral metastasis, *n* (%)**	15 (9.4)	0 (0.0)	15 (11.1)	0.181

BMI, Body Mass Index; CAD, Coronary Artery Disease; DM, Diabetes Mellitus; ECOG, Eastern Cooperative Oncology Group; HT, Hypertension; ISUP, International Society of Urological Pathology.

**Table 2:** **Treatment Parameters, Inflammatory Indices, and Clinical Outcomes of Metastatic Hormone-Sensitive Prostate Cancer (mHSPC) Patients as Defined by the CALLY** **Index.**

Characteristic	Total (*n* = 159)	CALLY High > 0.58 (*n* = 24)	CALLY Low ≤ 0.58 (*n* = 135)	*p*-Value
**Laboratory Parameters**				
Baseline PSA, ng/mL, median (IQR)	71.3 (20.2–312.0)	16.4 (10.7–83.4)	86.1 (26.8–314.0)	**0.003**
PSA nadir, ng/mL, median (IQR)	0.14 (0.01–1.68)	0.04 (0.01–0.41)	0.18 (0.02–2.00)	0.084
**First-Line ARPI Treatment**				
Enzalutamide, *n* (%)	78 (49.1)	11 (45.8)	67 (49.6)	0.300
Abiraterone, *n* (%)	71 (44.7)	13 (54.2)	58 (43.0)	
Apalutamide, *n* (%)	10 (6.3)	0 (0.0)	10 (7.4)	
**Treatment Response** (Best Overall Response)				
CR, *n* (%)	18 (11.3)	10 (41.7)	8 (5.9)	**<0.001**
PR, *n* (%)	133 (83.6)	14 (58.3)	119 (88.1)	
SD, *n* (%)	4 (2.5)	0 (0.0)	4 (3.0)	
PD, *n* (%)	4 (2.5)	0 (0.0)	4 (3.0)	
**Inflammatory Indices** **Median [IQR]**				
SII	653.8 [360–1003]	273.4 [178–369]	726.0 [461–1053]	**<0.001**
NLR	2.7 [1.8–3.6]	1.2 [0.8–1.6]	2.9 [2.0–3.9]	**<0.001**
SIRI	1.6 [1.0–2.5]	0.9 [0.5–1.2]	1.8 [1.1–2.6]	**<0.001**
**Outcomes**				
Disease progression, *n* (%)	51 (32.1)	4 (16.7)	47 (34.8)	0.129
Death, *n* (%)	41 (25.8)	2 (8.3)	39 (28.9)	0.062

Note: All 159 patients (100%) received continuous androgen deprivation therapy (ADT) as background therapy. CR, Complete response; IQR, Interquartile Range, NLR, Neutrophil-to-Lymphocyte Ratio; PD, Progressive disease, PR, Partial response; PSA, Prostate-Specific Antigen; SII, Systemic Immune-Inflammation Index; SIRI, Systemic Inflammation Response Index; SD, Stable disease.

**Table 3:** **Univariate and Multivariate Cox Proportional Hazards Analysis of Overall Survival** **(OS).**

Variable	Univariate Analysis HR (95% CI)	*p*-Value	Multivariate Analysis HR (95% CI)	*p*-Value
**CALLY Group**				
High (>0.58)	Reference	—	Reference	—
Low (≤0.58)	5.20 (1.25–21.6)	**0.021**	2.15 (0.52–8.90)	0.290
**CHAARTED Volume**				
Low	Reference	—	Reference	—
High	4.87 (1.90–12.4)	**0.001**	4.47 (1.74–11.4)	**0.002**
**Gleason Score**				
Continuous Scale	1.89 (1.13–3.17)	**0.014**	1.83 (1.08–3.09)	**0.024**
**Age**				
>65 vs. ≤65 years	1.39 (0.71–2.71)	0.338	1.85 (0.82–4.15)	0.140

CALLY, C-reactive protein–albumin–lymphocyte index; CI, Confidence Interval; HR, Hazard Ratio; Note: Multivariate model was adjusted for age, Gleason score, CHAARTED volume, and CALLY index. Note: The multivariable model included CALLY index, CHAARTED volume, Gleason score, and age as covariates (EPV = 41/4 = 10.25). CHAARTED volume and Gleason grade were selected for established prognostic relevance; age (>65 years) was included as an a priori confounder. LATITUDE risk was excluded due to collinearity with CHAARTED volume (shared defining parameters: bone lesion count, Gleason grade, and visceral metastasis). The proportional hazards assumption was tested using Schoenfeld residuals (global test: χ^2^ = 4.567, df = 4, *p* = 0.335; Supplementary Figure S3; individual covariates: CALLY *p* = 0.828, CHAARTED *p* = 0.753, Gleason *p* = 0.068, Age *p* = 0.628). The Gleason grade group showed a borderline non-significant trend in the Schoenfeld residual test (*p* = 0.068); however, the global test was clearly non-significant (*p* = 0.335), supporting retention of the standard Cox model. Apparent C-index = 0.696 (SE = 0.035).

**Table 4:** **Comparative Predictive Accuracy of Immune-Inflammatory and Nutritional Indices for Overall Survival** **(OS).**

Index	Cutoff	AUC	95% CI
**CALLY Index**	0.58	0.558	0.458–0.658
**SII**	507.4	0.534	0.428–0.639
**SIRI**	2.74	0.522	0.422–0.621
**NLR**	4.11	0.511	0.415–0.607

AUC, Area Under the Curve; CALLY, C-reactive protein–albumin–lymphocyte index; CI, Confidence Interval; NLR, Neutrophil-to-Lymphocyte Ratio; SII, Systemic Immune-Inflammation Index; SIRI, Systemic Inflammation Response Index. AUC values and 95% CIs are presented descriptively. Discriminatory ability was modest across all indices; no marker achieved an AUC clearly above 0.50. The log-rank analysis comparing Low- vs. High-CALLY groups is reported in Section 3.2.

## Data Availability

The data presented in this study are available from the corresponding author upon reasonable request. The data are not publicly available due to privacy and ethical restrictions.

## Supplementary Materials

The following supporting information can be downloaded at https://www.mdpi.com/article/10.3390/diagnostics16132126/s1, Figure S1: Original four-curve ROC plot comparing the discriminatory ability of CALLY, SII, SIRI, and NLR for overall survival (OS); Figure S2: Natural cubic spline model illustrating the non-linear relationship (*p* = 0.920) between continuous CALLY index and the log-relative hazard of overall survival (OS); Figure S3: Scaled Schoenfeld residuals plot for the multivariable Cox model testing the proportional hazards assumption over time; Figure S4: Distribution of the Youden-derived optimal CALLY cutoff across 1000 bootstrap resamples; Table S1: Sensitivity analyses and bootstrap internal validation results.

## References

[1] Siegel R.L., Kratzer T.B., Wagle N.S., Sung H., Jemal A. (2026). Cancer statistics, 2026. CA Cancer J. Clin..

[2] Goto T., Mizuno K., Sumiyoshi T., Kita Y., Masui K., Ogata T., Aizawa R., Sawada A., Saito R., Mizowaki T. (2026). Primary site- and metastasis-directed therapies in patients with metastatic prostate cancer: A narrative review. Int. J. Clin. Oncol..

[3] Marulanda-Corzo V., Holmes A.N., Giri V.K., Osborne J.R., Tagawa S.T. (2026). How will PSMAddition impact the management of prostate cancer?. Eur. Urol. Focus.

[4] Uzun M., Gokcek S., Kaya E., Semiz H.S. (2025). The prognostic role of systemic immune-inflammation index, SII, in Metastatic Castration-Resistant Prostate Cancer patients. Discov. Oncol..

[5] Sciarra A., Gentilucci A., Salciccia S., Pierella F., Del Bianco F., Gentile V., Silvestri I., Cattarino S. (2016). Prognostic value of inflammation in prostate cancer progression and response to therapeutic: A critical review. J. Inflamm..

[6] de Bono J.S., Guo C., Gurel B., De Marzo A.M., Sfanos K.S., Mani R.S., Gil J., Drake C.G., Alimonti A. (2020). Prostate carcinogenesis: Inflammatory storms. Nat. Rev. Cancer.

[7] Wang Z., Liu H., Zhu Q., Chen J., Zhao J., Zeng H. (2024). Analysis of the immune-inflammatory indices for patients with metastatic hormone-sensitive and castration-resistant prostate cancer. BMC Cancer.

[8] Iida H., Tani M., Komeda K., Nomi T., Matsushima H., Tanaka S., Ueno M., Nakai T., Maehira H., Mori H. (2022). Superiority of CRP-albumin-lymphocyte index (CALLY index) as a non-invasive prognostic biomarker after hepatectomy for hepatocellular carcinoma. HPB.

[9] Feng J., Wang L., Yang X., Chen Q. (2024). Clinical significance of preoperative CALLY index for prognostication in patients with esophageal squamous cell carcinoma undergoing surgery. Sci. Rep..

[10] Nakashima K., Haruki K., Kamada T., Takahashi J., Tsunematsu M., Ohdaira H., Furukawa K., Suzuki Y., Ikegami T. (2024). Usefulness of the C-Reactive Protein (CRP)-Albumin-Lymphocyte (CALLY) Index as a Prognostic Indicator for Patients with Gastric Cancer. Am. Surg..

[11] Sweeney C.J., Chen Y.-H., Carducci M., Liu G., Jarrard D.F., Eisenberger M., Wong Y.-N., Hahn N., Kohli M., Cooney M.M. (2015). Chemohormonal Therapy in Metastatic Hormone-Sensitive Prostate Cancer. N. Engl. J. Med..

[12] Fizazi K., Tran N., Fein L., Matsubara N., Rodriguez-Antolin A., Alekseev B.Y., Özgüroğlu M., Ye D., Feyerabend S., Protheroe A. (2017). Abiraterone plus Prednisone in Metastatic, Castration-Sensitive Prostate Cancer. N. Engl. J. Med..

[13] James N.D., Tannock I., N’DOw J., Feng F., Gillessen S., Ali S.A., Trujillo B., Al-Lazikani B., Attard G., Bray F. (2024). The Lancet Commission on prostate cancer: Planning for the surge in cases. Lancet.

[14] Rani A., Dasgupta P., Murphy J.J. (2019). Prostate Cancer: The Role of Inflammation and Chemokines. Am. J. Pathol..

[15] Michels N., van Aart C., Morisse J., Mullee A., Huybrechts I. (2021). Chronic inflammation towards cancer incidence: A systematic review and meta-analysis of epidemiological studies. Crit. Rev. Oncol..

[16] Shaul M.E., Fridlender Z.G. (2018). Cancer-related circulating and tumor-associated neutrophils—Subtypes, sources and function. FEBS J..

[17] Patrzałek P., Froń A., Mielczarek M., Karwacki J., Lesiuk G., Janczak D., Nagi K., Krajewski W., Dębiński P., Szydełko T. (2025). Inflammatory-based prognostic indicators in prostate cancer: Evaluating NLR, PLR, and SII in relation to Cambridge and ISUP classifications. Front. Oncol..

[18] Hébuterne X., Lemarié E., Michallet M., de Montreuil C.B., Schneider S.M., Goldwasser F. (2014). Prevalence of malnutrition and current use of nutrition support in patients with cancer. J. Parenter. Enter. Nutr..

[19] Sheinenzon A., Shehadeh M., Michelis R., Shaoul E., Ronen O. (2021). Serum albumin levels and inflammation. Int. J. Biol. Macromol..

[20] Acar C., Yüksel H.Ç., Şahin G., Açar F.P., Gunenc D., Karaca B. (2025). Prognostic utility of the CALLY index in metastatic melanoma: Building a nomogram for patients on anti-PD-1 therapy. Clin. Transl. Oncol..

[21] Di Lorenzo G., Buonerba L., Ingenito C., Crocetto F., Buonerba C., Libroia A., Sciarra A., Ragone G., Sanseverino R., Iaccarino S. (2020). Clinical characteristics of metastatic prostate cancer patients infected with COVID-19 in South Italy. Oncology.

[22] Zhuang J., Wang S., Wang Y., Wu Y., Hu R. (2024). Prognostic Value of CRP–Albumin–Lymphocyte (CALLY) Index in Patients Undergoing Surgery for Breast Cancer. Int. J. Gen. Med..

[23] Mizota K., Kinoshita F., Giacomo B., Tokunaga T., Hashinokuchi A., Matsudo K., Nagano T., Kosai K., Akamine T., Fujishita T. (2025). The Prognostic Impact of C-Reactive Protein-Albumin-Lymphocyte Index (Cally Index) in Patients with Surgically Resected Non-Small-Cell Lung Cancer. Ann. Surg. Oncol..

[24] Yang M., Lin S.-Q., Liu X.-Y., Tang M., Hu C.-L., Wang Z.-W., Zhang Q., Zhang X., Song M.-M., Ruan G.-T. (2023). Association between C-reactive protein-albumin-lymphocyte (CALLY) index and overall survival in patients with colorectal cancer: From the investigation on nutrition status and clinical outcome of common cancers study. Front. Immunol..

[25] Tsai Y.-T., Ko C.-A., Chen H.-C., Hsu C.-M., Lai C.-H., Lee Y.-C., Tsai M.-S., Chang G.-H., Huang E.I., Fang K.-H. (2022). Prognostic value of CRP-Albumin-Lymphocyte (CALLY) index in patients undergoing surgery for oral cavity cancer. J. Cancer.

[26] Jiang T., Sun H., Xu T., Xue S., Xia W., Xiao X., Wang Y., Guo L., Lin H. (2024). Significance of pre-treatment CALLY score combined with EBV-DNA levels for prognostication in non-metastatic nasopharyngeal cancer patients: A clinical perspective. J. Inflamm. Res..

[27] Cetinayak H.O., Aydin B., Semiz V., Kutlu E.A., Basan U., Aksoy R.A. (2025). Prognostic value of the CALLY index in hypopharyngeal cancer treated with definitive chemoradiotherapy: A retrospective cohort study. Diagnostics.

[28] Buonerba C., Ferro M., Dolce P., Crocetto F., Verde A., Lucarelli G., Scafuri L., Facchini S., Vaia A., Marinelli A. (2020). Predictors of efficacy of androgen-receptor-axis-targeted therapies in patients with metastatic castration-sensitive prostate cancer: A systematic review and meta-analysis. Crit. Rev. Oncol..

