# Supplementary material for: Prognostic Impact of Immune-Inflammatory and Nutritional Indices in Metastatic Hormone-Sensitive Prostate Cancer
Source: Diagnostics (Basel). 2026 Jul 7;16(13):2126. doi: 10.3390/diagnostics16132126 (PMC13360616; doi:10.3390/diagnostics16132126)

Supplementary Materials

Supplementary Table S1. Sensitivity analyses and bootstrap internal validation results.

Analysis	Estimate	95% CI	p-value
Continuous (per 0.1-unit)—OS Univariate	0.915	0.822 - 1.019	0.107
Continuous (per 0.1-unit)—OS Multivariable	0.955	0.855 - 1.068	0.423
Continuous (per 0.1-unit)—PFS Univariate	0.933	0.862 - 1.009	0.084
Continuous (per 1-SD)—OS Univariate	0.275	0.057 - 1.319	0.107
NCS non-linearity test (LR, df=2)	0.166	df = 2	0.920
Bootstrap C-index (1,000 iterations)	0.595	0.497 - 0.686	—
Bootstrap cutoff median (Youden)	0.046	0.004 - 0.907	—

Supplementary Figure S1. Original four-curve ROC plot comparing the discriminatory ability of CALLY, SII, SIRI, and NLR for overall survival (OS).

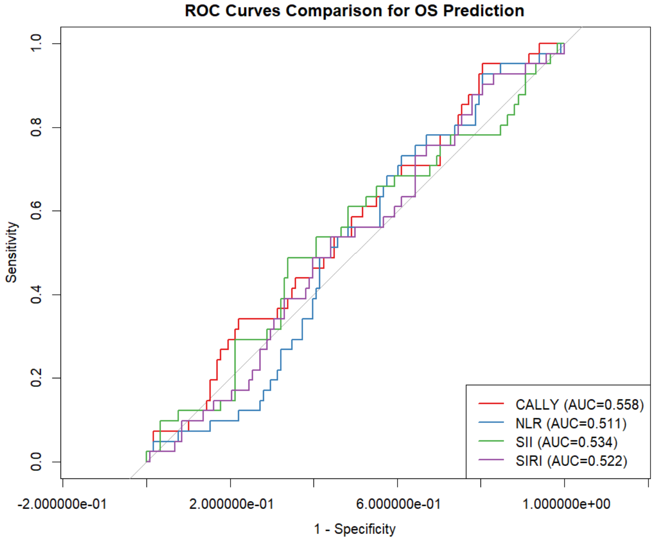

Supplementary Figure S2. Natural cubic spline model illustrating the non-linear relationship (p = 0.920) between continuous CALLY index and the log-relative hazard of overall survival (OS).

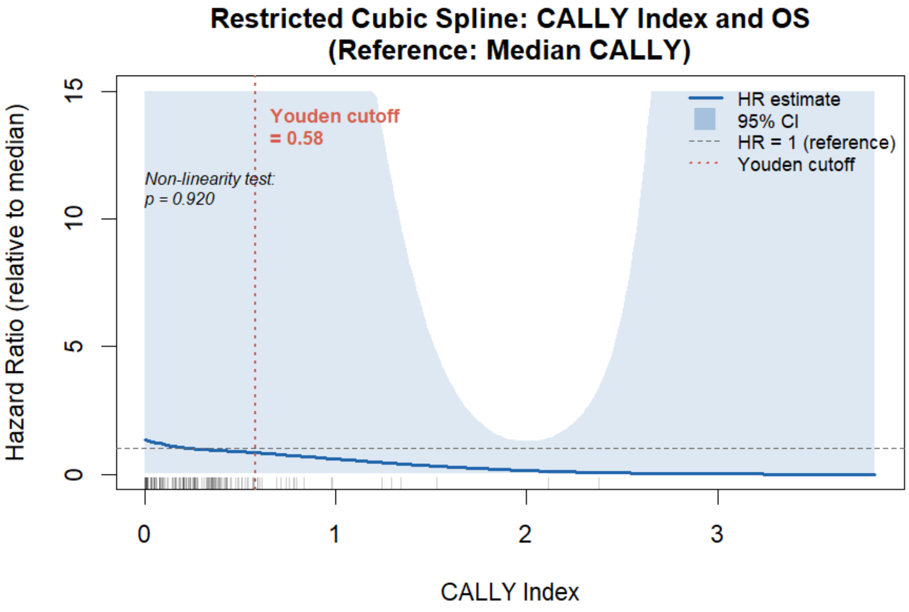

Supplementary Figure S3. Scaled Schoenfeld residuals plot for the multivariable Cox model testing the proportional hazards assumption over time.

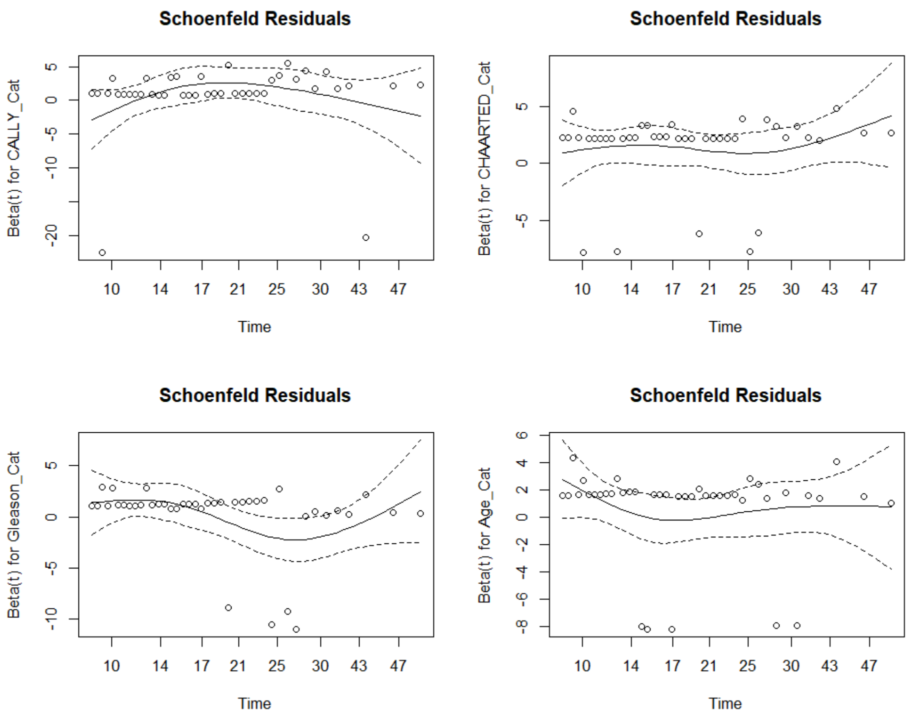

Supplementary Figure S4. Distribution of the Youden-derived optimal CALLY cutoff across 1000 bootstrap resamples.

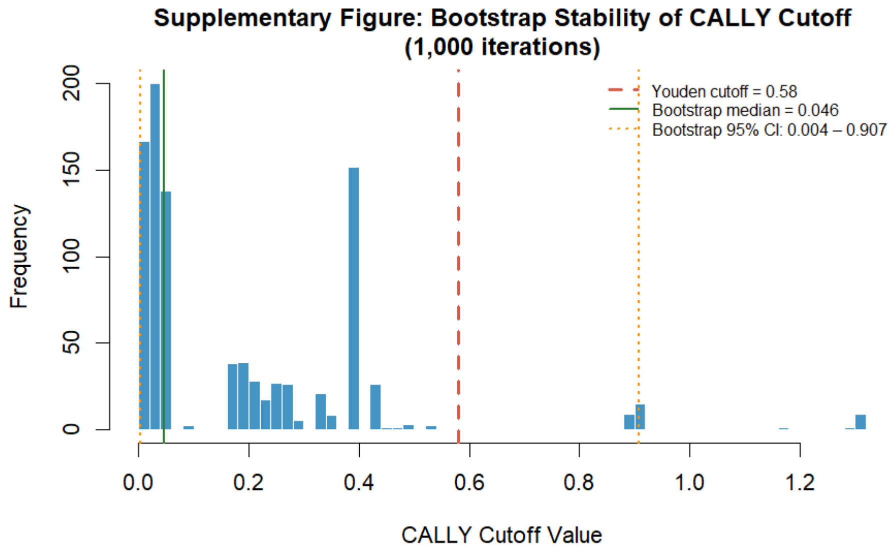

Supplement: Supplementary file 1 [file diagnostics-16-02126-s001.zip › diagnostics-4337974-supplementary.pdf]
